# Gastrointestinal stromal tumors of the upper GI tract: population-based analysis of epidemiology, treatment and outcome based on data from the German Clinical Cancer Registry Group

**DOI:** 10.1007/s00432-023-04690-6

**Published:** 2023-03-23

**Authors:** Thaer S. A. Abdalla, Lina Pieper, Markus Kist, Michael Thomaschewski, Monika Klinkhammer-Schalke, Sylke Ruth Zeissig, Kees Kleihues-van Tol, Ulrich Friedrich Wellner, Tobias Keck, Richard Hummel

**Affiliations:** 1 Department of Surgery, University Medical Center Schleswig-Holstein, Campus Lübeck, Ratzeburger Alle 160, 23564 Lübeck, Germany; 2 German Cancer Registry Group of the Society of German Tumor Centers - Network for Care, Quality and Research in Oncology (ADT), Berlin, Germany; 3 Institute of Clinical Epidemiology and Biometry (ICE-B), University of Würzburg, Würzburg, Germany

**Keywords:** Gastrointestinal stromal tumors, Esophagus, Gastroesophageal junction, Population-based

## Abstract

**Background:**

Gastrointestinal stromal tumors (GIST) are rare mesenchymal tumors. They are most frequently located in the stomach but are also found in the esophagus and the gastroesophageal junction (GEJ). Information regarding the prognostic factors associated with upper gastrointestinal GIST is still scarse.

**Methods:**

In this study, datasets provided by the German Clinical Cancer Registry Group, including a total of 93,069 patients with malignant tumors in the upper GI tract (C15, C16) between 2000 and 2016 were analyzed to investigate clinical outcomes of GIST in the entire upper GI tract.

**Results:**

We identified 1361 patients with GIST of the upper GI tract. Tumors were located in the esophagus in 37(2.7%) patients, at the GEJ in 70 (5.1%) patients, and in the stomach in 1254 (91.2%) patients. The incidence of GIST increased over time, reaching 5% of all UGI tumors in 2015. The median age was 69 years. The incidence of GIST was similar between males and females (53% vs 47%, respectively). However, the proportion of GIST in female patients increased continuously with advancing age, ranging from 34.7% (41–50 years) to 71.4% (91–100 years). Male patients were twice as likely to develop tumors in the esophagus and GEJ compared to females (3.4% vs. 1.9% and 6.7% vs. 3.4%, respectively). The median overall survival of upper gastrointestinal GIST was 129 months. The 1-year, 5-year, and 10-year OS was 93%, 79%, and 52% respectively. Nevertheless, tumors located in the esophagus and GEJ were associated with shorter OS compared to gastric GIST (130 vs. 111 months, p = 0.001). The incidence of documented distant metastasis increased with more proximal location of GIST (gastric vs. GEJ vs. esophagus: 13% vs. 16% vs. 27%) at presentation.

**Conclusion:**

GIST of the esophagus and GEJ are rare soft tissue sarcomas with increasing incidence in Germany. They are characterized by worse survival outcomes and increased risk of metastasis compared to gastric GIST.

**Supplementary Information:**

The online version contains supplementary material available at 10.1007/s00432-023-04690-6.

## Introduction

Gastrointestinal stromal tumors (GIST) are rare mesenchymal tumors that originate from the interstitial cells of Cajal [1]. They represent less than 1% of all neoplasms in the gastrointestinal tract. A global epidemiologic analysis demonstrated that most studies report an incidence of 10–15 per million per year (Søreide et al. 2016). The stomach is the most common site of GIST (60%), followed by the small intestines (35%), and lastly by the colon and rectum (5%). Less than 1% of tumors are found in the esophagus and gastroesophageal junction (Miettinen and Lasota 2006; Joensuu et al. 2013).

The prognosis of GIST depends on a variety of factors. Since 2001, many classifications have been proposed for the survival stratification of patients with GIST (Miettinen et al. 2005; Miettinen and Lasota 2006; Joensuu 2008; Gold et al. 2009; Agaimy 2010). Currently, the Armed Forces Institute of Pathology (AFIP) classification by Miettinen et al. is widely used and recommended by the ESMO guidelines (Casali et al. 2022). This classification uses location, size, and mitotic activity to estimate the risk of recurrence and progression. According to AFIP classification, gastric GISTs have a lower risk of recurrence or progression for the same size and mitotic activity compared to GIST of the small bowel and colon/rectum. Furthermore, positive resection margin and intraoperative tumor rupture are important negative prognostic parameters since they are associated with a higher likelihood of recurrence and poorer overall survival (Rutkowski et al. 2007; Takahashi et al. 2007).

Due to the low incidence of GIST in the esophagus and GEJ, information on their clinicopathological characteristics and clinical outcome are very limited. Most of the available literature on GIST focuses on the features of gastric and proximal small intestinal GIST, as these are the more common locations for GIST (Miettinen and Lasota 2006; Joensuu et al. 2013). As such, GIST of the esophagus and GEJ are not specifically addressed by the current classifications and specific treatment guidelines are lacking. In this context, the usage of big data sets such as population-based registries might represent a valuable source of information. Especially when targeting a subpopulation that is difficult to assemble by a single or small number of institutions. Therefore, this study aimed to evaluate the clinical features and outcomes of patients with GIST of the entire upper gastrointestinal (GI) tract including the esophagus, the GEJ, and the stomach based on a large data set of population-based clinical cancer registries in Germany provided by the German Cancer Registry Group of the Society of German Tumor Centers – Network for Care, Quality and Research in Oncology (ADT).

## Methods and materials

Data for the current study is derived from the German Cancer Registry Group of the Society of German Tumor Centers (ADT). The ADT aims to combine data from different German Clinical Cancer Registries all over the country on a voluntary basis. Hence, data for this study originates from 17 regional clinical cancer registries in 11 federal states. This study was approved by the local ethics committee of the University of Lübeck, Germany (20–327) and the ADT.

A total of 93,069 patients with a diagnosis of esophageal or gastric cancer (C15 and C16) were recorded between 2000 and 2016. Using the histological ICD-O-3 code “8936”, we identified approximately 1500 patients with gastrointestinal stromal tumors (GIST). After thorough preparation of the data set and testing for plausibility (e.g. exclusion of duplicates, exclusion of patients with the missing year of birth or diagnosis, exclusion of patients if the year of diagnosis was not recorded to be between 2000–2016, or exclusion of patients if the date of death was before or equal to the date of diagnosis), 1361 patients were finally included into the analysis (Fritz, Percy et al. 2000).

The following information was retrieved from the registry: sex, age at diagnosis (years), lymph node metastases (N0, N +), T-stage (T1–T4), Tumor location (esophagus (C15.0–15.9), cardia / GEJ (C16.0), stomach (C16.1–16.9)), type of therapy (none, operation alone, neoadjuvant systemic/chemotherapy/radiochemotherapy plus operation with/without adjuvant therapy, operation plus adjuvant systemic/chemotherapy/radiochemotherapy, systemic/chemotherapy/radiochemotherapy alone), operation type according to OPS-codes (partial resection of the esophagus (5–423; 5–424), total resection of the esophagus (5–426), local excision of the stomach (5–433), partial gastric resection (5–434, 5–435), subtotal gastrectomy (5–436), total gastrectomy (5–437), total gastrectomy with esophageal resection (5–438)), follow-up time (months after diagnosis), status at last follow-up (dead, alive).

### Statistical methods

For statistical analysis, SPSS 26 for Windows (Armonk, NY, USA) was used. Descriptive statistics were used to define patient baseline characteristics. To evaluate categorical variables, X^2^ test was applied. Survival curves for the overall survival of patients were plotted using the Kaplan–Meier method and compared using the log-rank test. Overall survival was computed as the period from the date of diagnosis to either the date of death or the last follow-up alive, whichever occurred first.. Patients alive at the last follow-up were censored for further analysis. Univariable cox regression analysis was used to determine prognostic variables for better or worse survival. For all statistical analyses, a p-value of p ≤ 0.050 was considered as significant.

## Results

### Patient characteristics and epidemiology

**Fig. 1:**
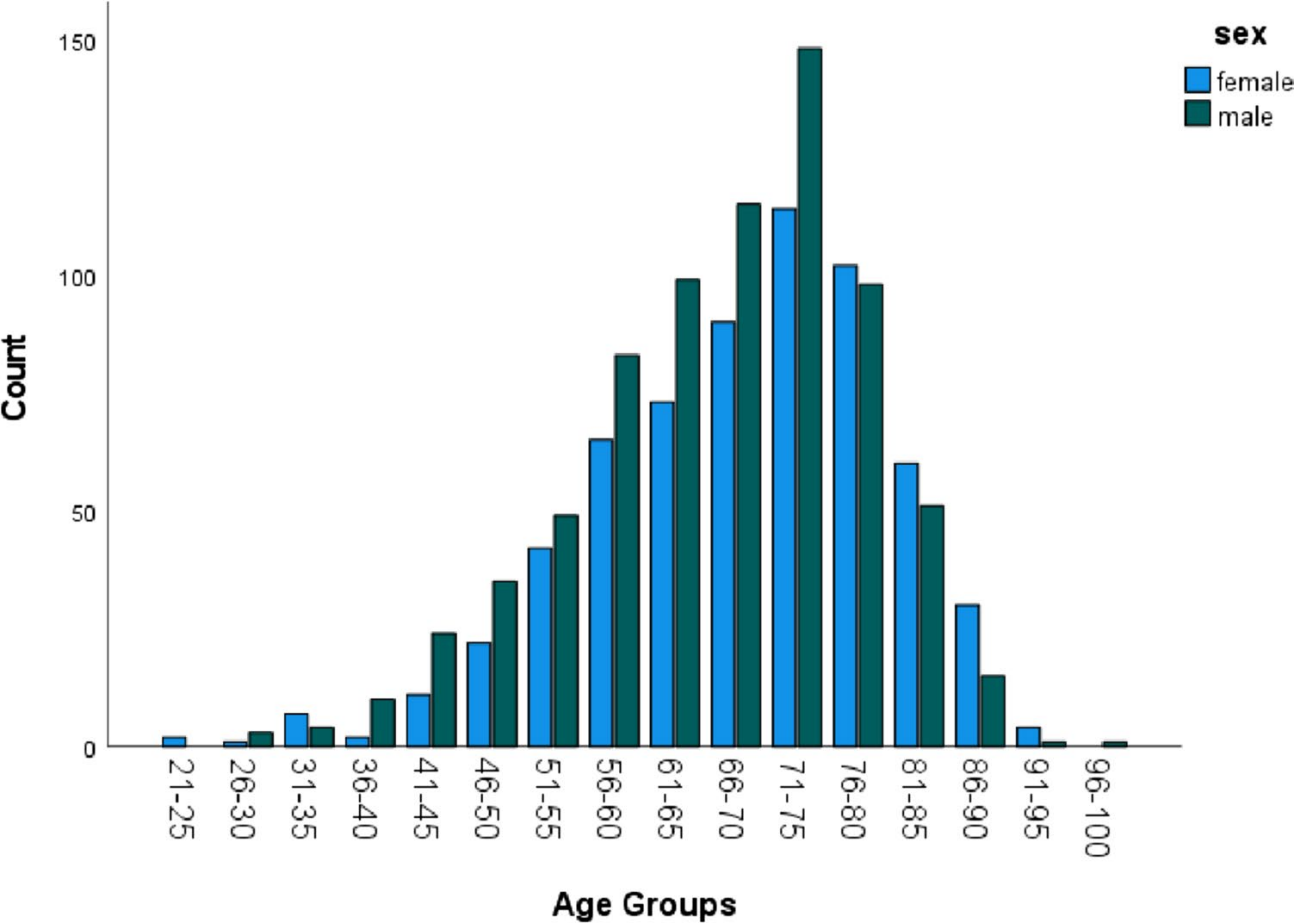
This figure shows the distribution of different age groups according to sex. Analysis of the reported cases showed a significant difference in the distribution of incidences between genders by age group. The proportion of females increased with increasing age, ranging from 37% (age group ≤ 50 years) to 44% (age group51-74 years) up to 53% (age group ≥ 75 years) (p < 0.001)

We identified 1361 patients with GIST in the upper gastrointestinal tract. 736 (54.1%) patients were male and 625 (45.9%) were female. The median age at diagnosis was 69 years (range 21–97 years). At the time of diagnosis, 1253 (92.1%) were 50 years or older. Only 25 patients (1.8%) were younger than 40 years. The proportion of females increased with increasing age, ranging from 37% (age group ≤ 50 years) to 44% (age group51-74 years) up to 53% (age group ≥ 75 years) (p < 0.001)(Fig. 1).

There was also an increase in the reporting rate of gastrointestinal stromal tumors relative to the total tumors diagnosed in the upper GI tract from 2000 to 2016. While the rate was 0.5% in 2000 and below 2% until 2007, it increased up to 4.9% in 2015.

### Tumor location of GIST in the upper GI tract

In the upper GI tract, 2.7% of all GIST were located in the esophagus (n = 37), 5.1% at the GEJ (n = 70), and 91.2% in the stomach (n = 1254). Gastric GIST was found in 38.7% of cases at an unspecified location and in 25.3% in the corpus. The most frequent location of GIST in the esophagus was the distal third (43.2%, n = 16).

In both men and women, the stomach was the main site of tumor manifestation (89.9% vs. 94.7%). However, men were almost twice as likely to present tumors in the esophagus (3.4% versus 1.9%) and the GEJ (6.7% versus 3.4%) compared to women (p = 0.005).

### Tumor size in GIST

Data regarding tumor size was available for 467 patients. In most patients, tumor size was > 2 to ≤ 5 cm (T2) at the time of diagnosis (n = 171, 36.6%). T1 tumors (≤ 2 cm) or T3 tumors (> 5 to ≤ 10 cm) were found at similar rates (n = 110, 23.6%, and n = 115, 24.6% respectively). T4 tumors (> 10 cm) were recorded in only n = 71 patients (15.2%). Interestingly, the predominant size of GIST in the different locations varied as esophageal lesions were mostly below 2 cm (T1; 53.3%), whereas GEJ and gastric tumors were mostly found at a size of > 2 to ≤ 5 cm (T2; 40.7% and 37.2%, respectively). However, tumor size did not differ significantly between groups (p = 0.125). Furthermore, the incidence of T3 and T4 tumors increased over time. While they were only sporadicly reported between the years 2001–2008, they represented almost one third of the cases after 2009. On the other hand, the proportion of T1 tumors reported in our cohort decreased slightly over time (Figure S1).

### Presence of distant metastasis in GIST

Distant metastases were documented in 186 patients (13.7%). Male gender was significantly associated with the occurrence of distant metastasis (male vs. female: 15.6% (n = 115) vs. 11.4% (n = 71); p-value 0.022). Furthermore, we found increasing rates of documented metastasis with advanced age (p =  < 0.001). Interestingly, only 4.8% (n = 9) of patients aged 31 to 40 years had documented metastatic disease at diagnosis, whereas 30.1% (n = 56) of patients aged 71 to 80 years presented documented metastatic disease.

The most frequently documented location of distant metastasis was the liver (n = 104; 68.4%), followed by the peritoneum (n = 25; 16.4%). Very rarely, distant metastasis occurred in organs such as the lung, pleura, or adrenal gland. While 27% of the patients with esophageal GIST were associated with metastasis at diagnosis, only 15.7% and 13.2% of patients with GEJ and gastric GIST reported to have distant metastasis at diagnosis.

**Fig. 2:**
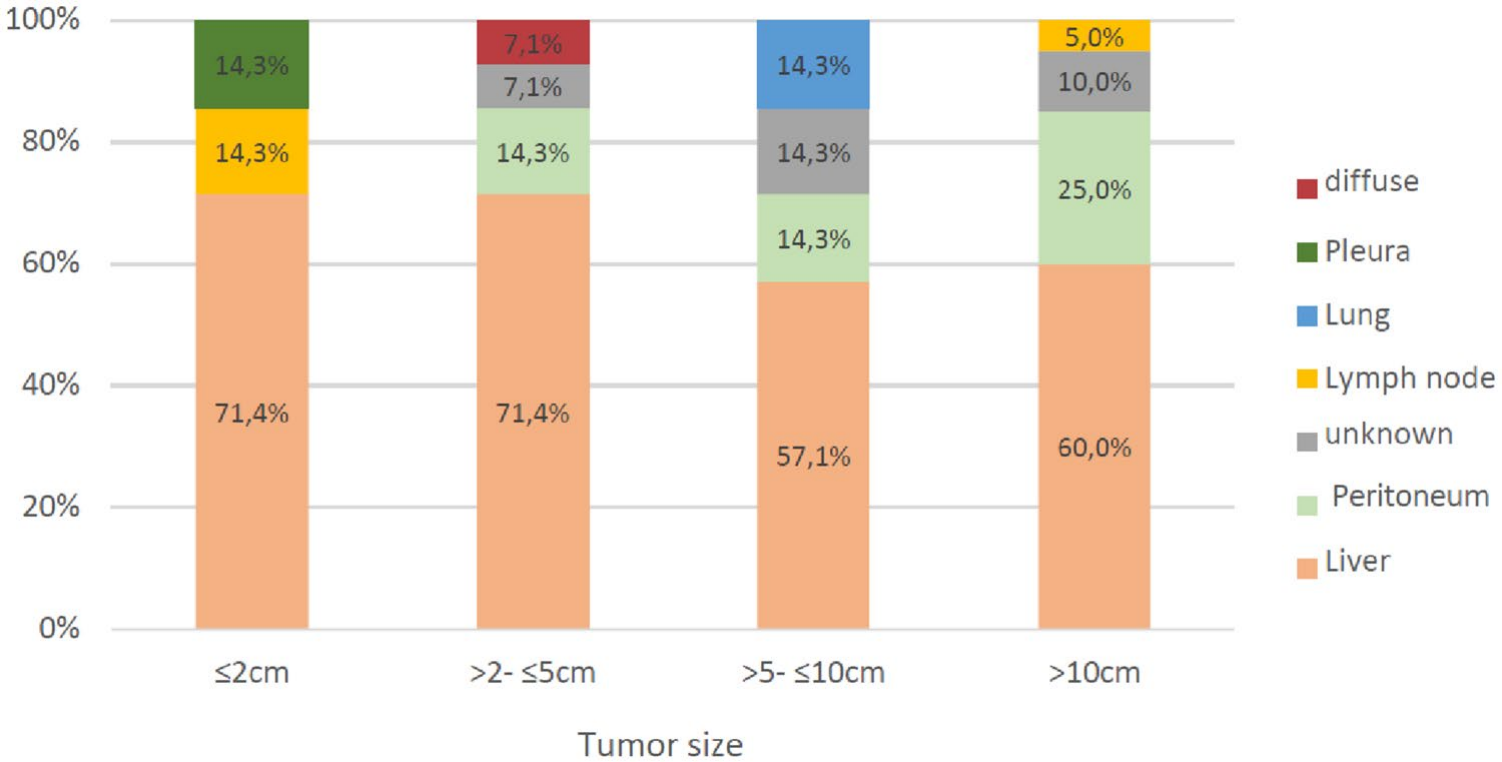
This figure shows the distribution of distant metastasis according to tumor size (n = 48, p = 0,506). The liver was the most common location for distant metastasis in GIST ≤ 2 cm in relation to other locations with a proportion of 71.4%. In GIST > 10 cm, liver metastasis was only reported in 60.0% of the patients. Conversely, peritoneal metastases were reported in 25.0% of GIST > 10 cm and decreased with decreasing tumor size

Furthermore, the incidence of documented metastasis was associated with larger tumors at diagnosis (p =  < 0.001). Most interestingly, we observed a trend toward the impact of tumor size on the location of metastasis. For example, the incidence of documented liver metastases was 71.4% in tumors ≤ 2 cm but decreased to 60% when the primary tumor size was larger than 10 cm. Conversely, documented peritoneal metastases were reported in 25% of tumors larger than 10 cm and decreased with smaller tumor sizes. However, this trend was not proven statistically significant (Fig. 2).

### Treatment

Detailed treatment information was available for 459 patients. Based on the intention of treatment, we divided the patients into”treatment with curative intention” (n = 234), "treatment with palliative intention” (n = 7), and "treatment with unknown intention” (n = 218). In the curative therapy approach, all patients received surgical intervention. In addition, 15.4% received additional drug therapy. Only seven patients were treated palliatively. In n = 218 patients, no information was available regarding the intention (curative or palliative). About 49.1% of these patients underwent surgery. The extent of surgery varied depending on tumor size, and tumor location. 70% of the patients received a partial gastrectomy, 24% required a more extensive gastric resection, and 6% required partial or total esophagectomy.

A total of n = 161 (11.8%) received systemic therapy. The majority of these patients (n = 114, 70.8%) received tyrosine kinase inhibitor treatment. The most common agent used in this context was imatinib with 67.0% (n = 108), followed by sunitinib or nilotinib.

### Survival analysis

Information regarding overall survival was available for 1249 patients. According to the Kaplan–Meier analysis, the median survival time of GIST patients was 129 months. Tumors located in the stomach were associated with longer median overall survival (130 months) compared to patients with GIST of the esophagus (97 months) or GEJ (72 months). The 1-year, 3-year, 5-year, and 10-year overall survival rates in patients with GIST of the upper GI tract were 93.2%, 85.8%, 78.9%, and 51.8% respectively.

**Fig. 3:**
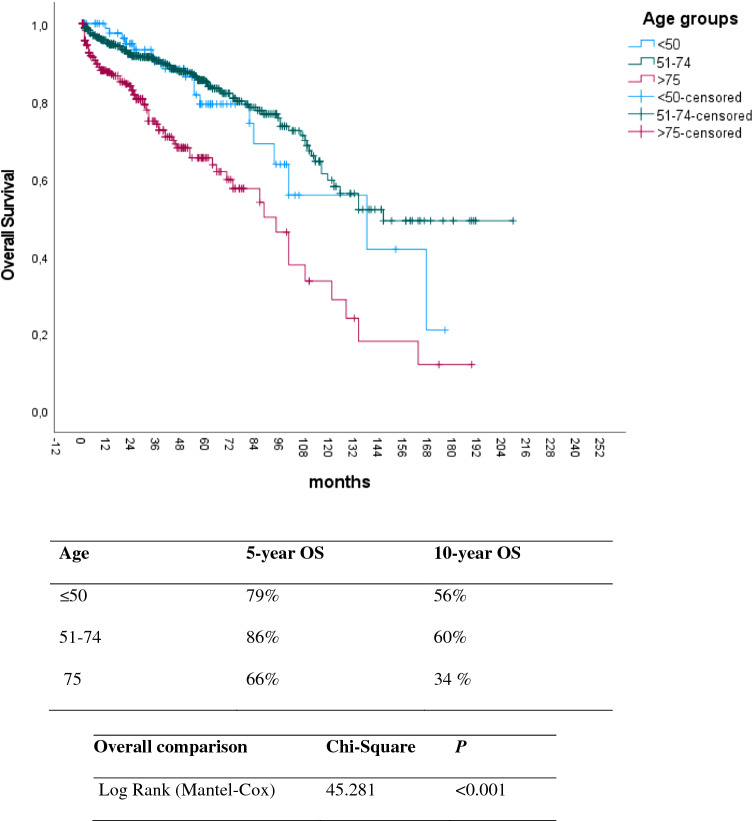
Kaplan–Meier plot for overall survival in patients with upper gastrointestinal GIST according to different age groups. Increasing age was associated with worse overall survival. p-value was estimated using the Log-Rank test

**Fig. 4:**
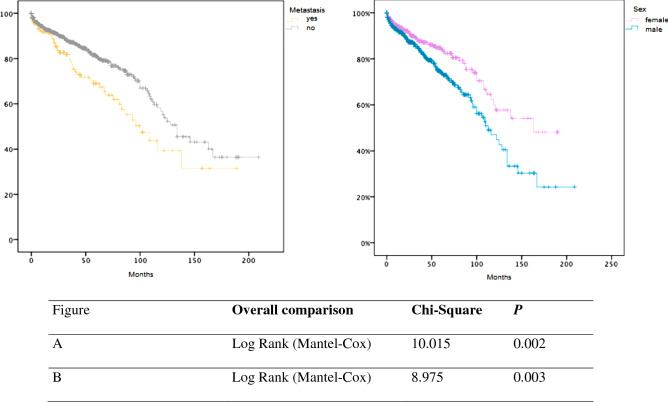
Figures A and B show the association of metastatic disease at diagnosis and gender with overall survival in patients with upper gastrointestinal GIST. The presence of metastasis and male gender were associated with poor survival (p-value = 0.003) and (p-value = 0.002), respectively. p-value was estimated using the Log-Rank test

**Fig. 5:**
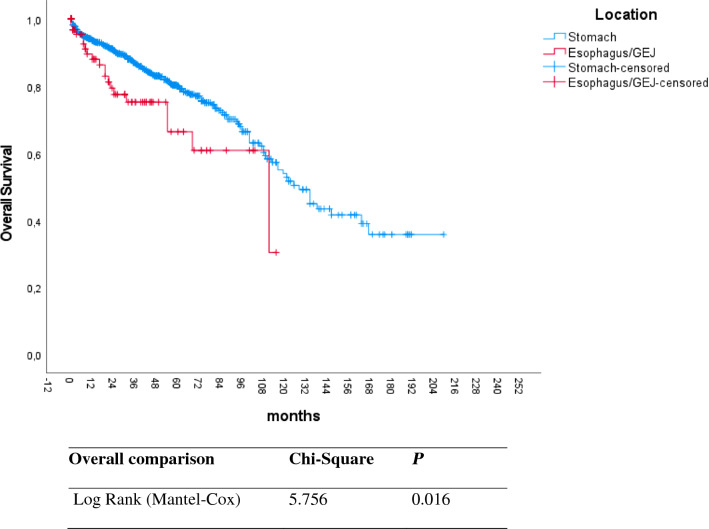
This Figure shows a Kaplan–Meier Plot for overall survival in patients with GIST according to tumor location. Patients with esophageal or GEJ GIST were associated with worse survival compared to patients with gastric GIST

The univariable regression analysis demonstrated that advanced age (p =  < 0.001), male sex (p = 0.002), presence of metastasis (p = 0.003), and tumor location in the esophagus or GEJ (p = 0.016) were associated with shorter overall survival (Figs. 3, 4 and 5) (Table 1).

**Table 1:** Univariable analysis of different factors and their impact on overall survival in GIST

Variables	p-Value	Hazard-Ratio (95% CI)	Median OS months
**Sex**			
Male	Reference		113
Female	0.002	0.618 (0.456–0.836)	163
**Age**			
> 75 years	Reference		92
51–74 years	< 0.001	0.366 (0.269–0.499)	151
< 50 years	< 0.001	0.453 (0.271–0.758)	137
**Location**			
Stomach	Reference		130
Esophagus or GEJ	0.018	1,75 (1.101–2.803)	111
**Size**			
≤ 2 cm	Reference		84
> 2- ≤ 5 cm	0.874	1.067 (0.479–2.376)	113
> 5- ≤ 10 cm	0.514	1.313 (0.579–2.975)	100
> 10 cm	0.872	1.080 (0.424–2.748)	92
**Presence of Metastasis**			
No Metastasis	Reference		134
Distant Metastasis	0.003	1.643 (1.182–2.283)	100

Legend: *p* Indicates significance according to cox regression analysis of the specified variables. HR indicates hazard ratio

## Discussion

Gastrointestinal stromal tumors of the esophagus and gastroesophageal junction (GEJ) are very rare tumors and have an estimated incidence of 0.1 to 0.3 per million people (Miettinen and Lasota 2006; Joensuu et al. 2013). Therefore, little is known about their clinicopathological features and clinical outcome. With this work, we present the first population-based analysis for GIST of the entire upper gastrointestinal tract, including esophageal, gastroesophageal junction, and gastric GIST using large population-based clinical cancer registry data provided by the German Cancer Registry Group of the Society of German Tumor Centers—Network for Care, Quality and Research in Oncology (ADT).

Our results showed a steady increase in the reported diagnosis of gastroesophageal GIST for the period 2000–2016, reaching a peak of 5% of all upper GI tumors in our registry in 2015. In addition, we found that GIST of the esophagus and GEJ are rare tumors, compared to gastric GIST representing 0.04% and 0.08% of all upper GI tumors as well as 2.7% and 5.1% of all reported GIST in our cohort. These observations are consistent with other population-based studies (Miettinen et al. 2005; Tran et al. 2005; Briggler et al. 2018). The reasons for this internationally observed increase in the rate of new cases are manifold. Firstly, the diagnostic criteria have changed over time. For example, the immunohistochemical markers CD117 (KIT) and DOG1 became standard in routine diagnostics, accompanied by an increasing awareness of the disease among physicians. Epidemiologic cancer registries had long included only GIST coded as malignant; that is, only tumors with ICD-O codes 8931/3 or 8936/3 were included in incidence estimates. However, the latest update of the WHO classification for sarcomas now classifies all GISTs as "malignant," regardless of tumor size, location, or mitotic rate (Cancer 2020). Another possible reason might be an improved surveillance and endoscopy rate among the general population in germany in the last decades(Rey, Hoffman et al. 2014). However, information on surveillance or increased use of endoscopy can not be analysed using the present dataset.

Furthermore, our data revealed that the incidence of GIST was higher in male compared to female patients younger than 75 years, but was subsequently higher in fmela compared to male patients at an age > 75 years (Fig. 1). This observation might be biased by the current longer life expectancy of females in the general population worldwide (Kontis et al. 2017). In addition, we demonstrated in the univariable analysis that male patients have shorter median OS compared to female patients (165 vs 115 months, p = 0.003). As suggested by Jzerman et al. this might be due to the more aggressive tumor characteristics at baseline such as higher mitotic and increased tumor rupture in male patients(NS, van Werkhoven et al. 2022). Nevertheless, in our cohort, the available baseline characteristics like tumor size and presence of metastasis were not associated with sex differences in patients with gastroesophageal GISTs.

Moreover, GIST of the esophagus or GEJ were associated with shorter median overall survival when compared to gastric GIST (111 months vs. 130 months, p = 0. 016 respectively). More importantly, these tumors also demonstrated increased metastatic potential compared to gastric GIST. Specifically, distant metastasis was documented in 27% of patients with esophageal GIST compared to only 15.7% and 13.2% of patients with GEJ and gastric GIST (p = 0.047). Similar results were reported by Theiss et al. where the incidence of metastases in esophageal GIST ranged between 17.0% to 48.0%(Theiss and Contreras 2019). Although more than half of the tumors in the esophagus were small (≤ 2 cm), esophageal tumors represented the subgroup with the greatest metastatic potential. As previously suggested, this phenomenon could be explained by the lack of serosal covering of the esophagus which can propagate its metastatic potential (Tran et al. 2005; Lott et al. 2015; Briggler et al. 2018). Until now, tumor location of GIST in the esophagus and GEJ have not been specifically addressed by any of the GIST risk stratification classifications, including AFIP, and Joensuu criteria. Nevertheless, our data suggest that esophageal and GEJ GIST can be classified into a higher-risk category.

Furthermore, it is well known that gastroesophageal GIST occurs in up to > 80% of cases in adults older than 50 years (Mucciarini, Rossi et al. 2007, Joensuu et al. 2013). This was reflected in our data, in fact, 92.1% of all patients were ≥ 50 years of age and only 1.8% were younger than 40 years of age at the time of diagnosis.

Approximately 13.7% of patients had documented metastasis at diagnosis. Incidence of metastasis was associated with advanced age, tumor size, and with tumors located in the esophagus. While tumors ≤ 2 cm were only in 12.9% of the patients associated with documented distant metastases, those larger than 10 cm developed metastasis in 35.7% of the patients. Similar results were found in the literature (DeMatteo et al. 2000; Trupiano et al. 2002). Interestingly, tumor size showed no significant impact on survival in our cohort and the respective survival rates did not show any major differences in the first five years. This is surprising as tumor size has been shown in several studies to play an important prognostic role concerning malignancy and the tendency to distant metastasis of the GIST (DeMatteo et al. 2000; Trupiano et al. 2002). However, it should be noted that the mentioned studies were performed before or in the initial period of imatinib. As previously described, the prognosis of GIST has significantly improved since the introduction of imatinib. (Perez et al. 2006; Cavnar et al. 2021). Thus, tumor biology cannot be determined on the basis of tumor size alone, but other factors such as mitotic activity or mutation analysis should be taken into consideration. Especially, since activating mutations in KIT proto-oncogene receptor tyrosine kinase or in platelet-derived growth factor alpha are considered major drivers of GIST and their detection predicts treatment responsiveness or resistance (Joensuu et al. 2017). For example, the presence of PDGFRA mutation predicts resistance to Imatinib or the presence of KIT exon 9 mutation stratifies the patients who will benefit from higher doses of Imatinib(Debiec-Rychter et al. 2006). Unfortunately, mitotic activity and mutation analysis are not documented in the data set used for this analysis, hence we cannot provide an informative analysis of this aspect of tumor biology.

The organs affected by distant metastasis were predominantly the liver and the peritoneum (68.4% and 16.4%, respectively), Interestingly, we noticed a change in metastatic patterns according to the tumor size. While the percentage of liver metastases decreased with an increase in tumor size, the percentage of peritoneal metastasis increased. Similarly, Yang et al. reported that the incidence of liver metastases was associated with increasing tumor size (Yang et al. 2019).

In addition, we demonstrate that patients with gastroesophageal GIST have a 5- and 10-year overall survival of 78.9% and 51.8%. Interestingly, even the presence of metastasis at presentation was associated with relatively long 5- and 10-year overall survival rates of 69% and 39.4% and a median OS of 100 months, which is two to fivefold higher than in previously published results by Ma et al. which included patients with liver, bone, and lung metastatic GIST. Moreover, longer overall survival was associated with female gender, age < 75 years, gastric GIST, and absence of distant metastases.

Although this study represents a large population-based registry study, several limitations must be addressed for the proper interpretation of results. First, the German Clinical Cancer Registry Group collects data from several regional clinical cancer registries on a voluntary basis, implicating data entry by many different people in variable quality. In addition, several variables were not included in the predefined dataset, such as the mitosis rate and mutation analysis (like KIT and PDGFRA mutation), therefore the effect of these variables on patients’ survival could not be assessedand our analysis could not be adjusted for potential confounding factors. Another limitation, is that a multivariable analysis was not conducted due to variability in completeness of some variables and the consequently low number of patients among the different subgroups. Some variables, such as disease-free survival, were not analyzed due to a large number of missing information. Information regarding response to medical treatment was not available in our dataset. On the other hand, due to the large number of patients in our cohort, we were able to provide a representative overall picture of GIST arising in the upper GI tract and to describe the daily clinical practice in Germany. To our knowledge, only a few publications to date exist with a comparable caseload, emphasizing the urgent need for more research and the creation of GIST-specific registries.

## Conclusion

This is the largest population-based study on the incidence and characteristics of GIST of the entire upper gastrointestinal tract (esophagus, GEJ, and stomach). Our results demonstrate that the proportion of female patients in the GIST population increased with increasing age and that male patients are twice as likely to develop tumors in the esophagus and the GEJ compared to females. Moreover, tumors in GEJ and esophagus are associated with shorter overall survival and increased metastatic potential compared to those in the stomach, which justifies in our opinion classification of this specific group of patients into a high-risk category.

## Supplementary Information

Below is the link to the electronic supplementary material.

Supplementary file1 (DOCX 123 kb)

## Data Availability

Data were obtained from The Society of German Tumor Centers (Arbeitsgemeinschaft Deutscher Tumorzentren, ADT) and are available from the authors with the permission of The Society of German Tumor Centers.
