# Supplementary material for: Gastrointestinal stromal tumors of the upper GI tract: population-based analysis of epidemiology, treatment and outcome based on data from the German Clinical Cancer Registry Group
Source: J Cancer Res Clin Oncol. 2023 Mar 23;149(10):7461–9. doi: 10.1007/s00432-023-04690-6 (PMC10374476; doi:10.1007/s00432-023-04690-6)
Supplement: Supplementary file 1 — Supplementary file1 (DOCX 123 kb) [file 432_2023_4690_MOESM1_ESM.docx]

Supplementary Material:


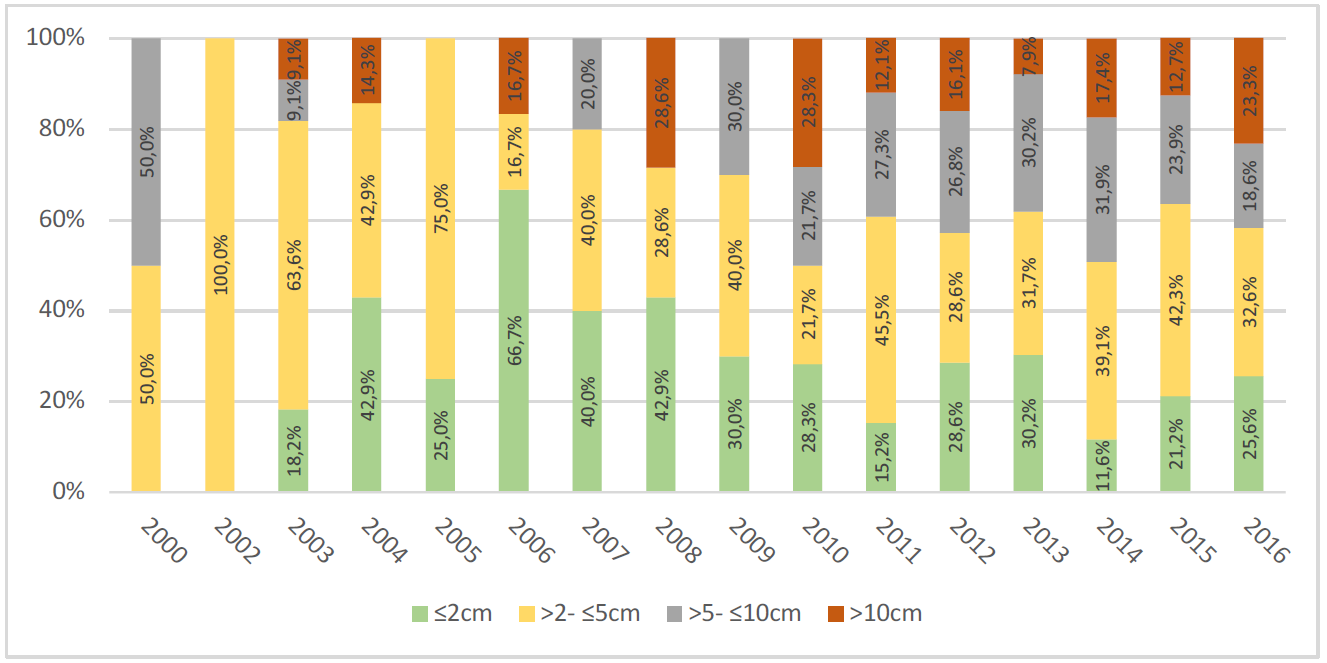
Figure S1: This figure demonstrates the changes in tumor size of reported upper gastrointestinal GIST over period (2000-2016). (n=467, p-value=0.086 according to X^2^ Test).
